# Photosynthesis research: a model to bridge fundamental science, translational products, and socio-economic considerations in agriculture

**DOI:** 10.1093/jxb/eraa087

**Published:** 2020-02-19

**Authors:** Ajay Kohli, Berta Miro, Jean Balié, Jacqueline d’A Hughes

**Affiliations:** 1 International Rice Research Institute, Los Baños, Philippines; 2 Research School of Biology, Australian National University, Australia

**Keywords:** C_3_–C_4_, climate change, nutritious crops, photosynthesis, rice, sustainability, socio-economic frontier, translational products, yield

## Abstract

Despite impressive success in molecular physiological understanding of photosynthesis, and preliminary evidence on its potential for quantum shifts in agricultural productivity, the question remains of whether increased photosynthesis, without parallel fine-tuning of the associated processes, is enough. There is a distinct lack of formal socio-economic impact studies that address the critical questions of product profiling, cost–benefit analysis, environmental trade-offs, and technological and market forces in product acceptability. When a relatively well understood process gains enough traction for translational value, its broader scientific and technical gap assessment, in conjunction with its socio-economic impact assessment for success, should be a prerequisite. The successes in the upstream basic understanding of photosynthesis should be integrated with a gap analysis for downstream translational applications to impact the farmers’ and customers’ lifestyles and livelihoods. The purpose of this review is to assess how the laboratory, the field, and the societal demands from photosynthesis could generate a transformative product. Two crucial recommendations from the analysis of the state of knowledge and potential ways forward are (i) the formulation of integrative mega-projects, which span the multistakeholder spectrum, to ensure rapid success in harnessing the transformative power of photosynthesis; and (ii) stipulating spatiotemporal, labour, and economic criteria to stage-gate deliverables.

## Teaser

Achievements in photosynthesis research portend a capacity for transformative changes in food and nutritional security if integrated with water and nutrient use and the requirements of multiple stakeholders along the value chain.

## Introduction

The human population grew from 1 to 2 billion in 123 years but from 5 to 6 billion in only 12 years (1987–1999), and, again in 12 years (1999–2011), from 6 to 7 billion (BBC News, https://www.bbc.com/news/world-15459643). Between 2025 and 2030, the global population is set to increase by at least another billion and may exceed 10 billion in around 2050 (UNDESA, https://www.un.org/development/desa/en/news/population/world-population-prospects-2019.html). Although population increase is the main reason to consider ways and means for producing more food, other critical factors necessitate a reappraisal of the agricultural practices, processes, and production. For example, the decreasing availability, accessibility, and affordability of one or more components of the land–water–labour–energy nexus affect agriculture globally. Similarly, the demand for increased production of seeds superior in agronomic and/or nutrition performance calls for paradigm shifts from increasing productivity for cheap food to sustainably intensifying agriculture to provide healthy food.

Asia is home to nearly 60% of the world’s population. Hence rice, Asia’s staple food, would be a target crop of high priority for research on increasing quantity and quality. Rice suffers more than the other crops from decreasing agricultural land, water, and labour, and is energy intensive. Also, its cultivation practices account for greater emission of greenhouse gases (GHGs), such as methane and nitrous oxide, than other cereals. It is particularly sensitive to other stresses such as drought, heat, cold, flooding, and salinity. Unlike wheat and maize, rice lacks the ‘futures market’ which underlies a comparatively less organized national and international rice seed industry. Consequently, the rice seed quality at input and its consequent output can suffer.

Rice is not only the staple food and lifeline for the majority of the world’s poor and hungry, but it is also key to improving economic stability and quality of life in most Asian countries due to national and international market and socio-economic forces. The demand for rice cultivation and consumption in Africa is also steadily increasing. These factors, when taken together, argue for improvements in productivity and quality, and in sustainable intensive cultivation, of rice in Asia and Africa.

Research agendas that address the increase in quantity and quality of rice and other crops for the future must be harmonized for a united global community effort to develop and utilize technological resources (Zaidi *et al.*, 2019). Sufficient and nutritious food for the future is a global imperative that cannot be advanced sustainably without global transdisciplinary efforts. For example, problems in agriculture, associated with climate change, are still patchily understood. They are primarily geopolitical and/or ecoregional, and are currently addressed through local or regional solutions. Only with a global approach to the problems and solutions can sustainable productivity be achieved. Regional sea level rise and consequent salination, acidification, or eutrophication can be ameliorated for the long term if global players agree to support reduction of emissions, soil reclamation, availability of adapted varieties, and perhaps even consider relocation. Research on reduction of agricultural emissions on the global scale and salt-tolerant crop varieties at the regional scale, for example, can be helpful.

There are three major and interconnected research routes for increasing crop productivity for the future scenarios: phenotyping-based, genotyping-based, and physiological process-based. Historically, the phenotyping-based route would be the classical breeding and agronomy approach, and contemporarily the genotyping-based route would include all molecular approaches including various ‘omics’ methods of characterizing a genotype. However, the physiological process-based route would be the modern molecular physiology approach. Nevertheless, under the modern context of generating agricultural knowledge and products, the three routes cannot be independent of each other, and it is critical that the three routes are integrated to develop improved, adapted, productive, and consumer-preferred crop varieties. Keeping the farmers and consumers in mind, the need to maintain and increase nutritional quality applies to all cereals but is critical in rice for which the market forces work more around the grain attributes than around the flour attributes.

One particular plant trait, the understanding of which has progressed over time through an integrative course, is photosynthesis. Photosynthesis is the very basis of human existence. An extensive mechanistic understanding of this process over the years, from morphology and anatomy to biochemistry to molecular biology to biophysics and electrophysiology, makes photosynthesis an intuitively attractive and plausible avenue for increasing plant productivity. A relatively deep understanding of photosynthesis drives our efforts to manipulate it for increased crop productivity.

From the early to mid-18th century, discoveries revealed that plants derive nourishment from the atmosphere through their leaves and that the gas produced by plants is oxygen (Bonnet, 1754). Huzisige and Ke (1993) chronicled the timeline of landmark discoveries in understanding photosynthesis. That timeline revealed that studies in the 1830s and 1840s led to recognizing that light, chlorophyll, and chloroplasts are needed and that light energy converts into chemical energy. It took until the 1930s to determine that photosynthesis is a light-induced oxidation–reduction process and the Calvin–Benson cycle was elucidated only in the 1950s. Significant progress was made in biochemical and biophysical aspects underpinning photosynthesis in the 1960s. In the next decade, the carboxylase/oxidase functions of Rubisco (EC 4.1.1.39) were identified and the 3-D structures of Rubisco and mutants of Cyt *b* were available in the late 1980s and 1990s. Through the progressive mechanistic understanding of photosynthesis, the source–sink relationships between the photosynthetic assimilates also became clear (Ho *et al.*, 1983), as did the unique connection between the sink and crop improvement for human consumption.

In the 1990s, ideas were proposed to manipulate (and improve) photosynthesis as a means to further improve crops generated during the ‘Green Revolution’, but the yields of which had started to plateau (Janaiah *et al.*, 2006). Incremental success, since the Green Revolution, in improving the yield of cereals augmented crop productivity linearly by ~1.7% annually (Long *et al.*, 2015). However, substantial yield increase did not always accompany suitable grain quality, and varieties with extremely high yield potential were rejected, leading to recent efforts in designing strategies to increase yield and quality simultaneously (Zeng *et al.*, 2017). With molecular tools and plant transformation, the pipeline of gene identification, isolation, and characterization took centre stage. However, no single yield-related gene transformation led to a substantial yield increase. Gene combinations are starting to show promise, but this is only through traits of low heritability such as panicle number (Huang *et al.*, 2018). Photosynthesis is the only frontier that is central to plant growth, development, and yield; relatively well understood at the mechanistic level, yet not fully harnessed in the downstream application for increased crop productivity and quality.

## Can manipulation of photosynthesis lead to an increase in grain yield?

Atmospheric CO_2_ concentrations have risen by 30% over the last 60 years (https://www.esrl.noaa.gov/gmd/obop/mlo/), directly affecting photosynthesis. One of the predictions is an increase in yield of C_3_ plants by 2- or 3-fold. These predictions are based on studies that increased exogenous atmospheric CO_2_ by free-air CO_2_ enrichment (FACE; Ainsworth and Long, 2005; Gielen *et al.*, 2005). The biomass of woody trees (e.g. poplar) increased by 15–30%. The technique was then tested with crops, including cereals, in field conditions in a crop rotation of barley, rye, sugar beet, and winter wheat, and yields increased by ~12% in barley, wheat, and sugar beet (Weigel and Manderscheid, 2012). However, the limited numbers of FACE experiments with a limited number of crops show considerable seasonal, geographical, and genotype variability for an increase in biomass and yield. The study of Weigel and Manderscheid (2012) also suggested a positive effect of elevated CO_2_ on yield under drought and also concluded that the variation in nitrogen content did not affect yield. However, there is considerable variability in the positive effect of elevated CO_2_ on yield depending on the water and nitrogen content available. In a FACE study with rice under different levels of nitrogen, elevated CO_2_ caused a similar increase in biomass under three different nitrogen regimes, but the yield increase was proportional to the nitrogen content (Kim *et al.*, 2003). Similarly, the positive effects of elevated CO_2_ were more consistent in high-yielding cultivars with higher nitrogen use efficiency (NUE), thus once again highlighting the role of nitrogen (Hasegawa *et al.*, 2013). The importance of nitrogen in preference to photosynthesis as a route to increasing crop yield was recently discussed (Sinclair *et al.*, 2019). Vegetative biomass tends to increase during early growth and development under enhanced CO_2_ concentration, but this may not be maintained during reproductive development.

Most studies have assumed that with elevated CO_2_, a temperature increase occurs. At a lower temperature, the yield increase is much lower than at higher temperatures. Modelling studies predict that the beneficial effect of rising temperatures may not be sustained above certain temperature increases (Degener, 2015). Simultaneously, high temperature is known to negatively affect yield and grain quality (Krishnan *et al.*, 2011). Maize cultivation is already under optimal temperatures and could be negatively affected by higher temperatures (Schlenker and Roberts, 2009). FACE studies conducted over time and across space have not used the same elevated CO_2_ concentrations. Thus, the interplay between CO_2_ and temperature, and combined effects on yields, remains ambiguous. Deciphering dependable patterns from the limited number of studies appears difficult and also inconclusive due to the multiplicity of variables in nutrient, water, temperature, edaphic, and soil microbial factors and their interactions under altered CO_2_ concentrations. For example, the yield of maize, a C_4_ plant, is not predicted to react substantially to elevated CO_2_, although it may improve under water stress (Kimball, 2011). Such an indirect sustainability effect through water saving is thought to be of a considerable proportion and could be equally, if not more, useful than the benefits to agriculture through elevated CO_2_ (Morgan *et al.*, 2004). Also, recent FACE studies by Lv *et al.* (2020) revealed that temperature, nitrogen, and genotype variability have major contributions to the yield increase under FACE, once again stressing the interaction of multiple variables contributing to yield. Thus, modification of plants for improved photosynthesis, engineered or bred, for better capture or utilization of CO_2_ may not directly increase grain yield.

The prospects for the potential benefits to plant productivity from elevated CO_2_, as seen through FACE studies to date, raise some questions as well. First, considering the complex air–water–nutrient–soil–temperature nexus, how much of the beneficial effects of elevated CO_2_ are due to increased photosynthesis *per se*? In other words, do we have enough evidence that manipulation of the molecular and biochemical machinery of photosynthesis, to replicate the scenario of providing more CO_2_ to the plant, or a more efficient use or regeneration of CO_2_ inside the plant cells, leads to desirable levels of increase in agricultural productivity? Secondly, will the harvest index increase with the increase in photosynthesis; will the increase in biomass be preferentially partitioned to grain? Thirdly, with the possibility of the use of big data, machine learning, and artificial intelligence, would investments in the next-generation FACE studies be beneficial if they address the complex interactions among the critical variables of air, water, nutrients, soil, and temperature? Cai *et al.* (2016, 2018, 2020) conducted a series of studies of rice grown under FACE, showing that various biochemical and photosynthetic parameters were differentially affected by CO_2_ concentration, temperature, and growth stage. Such multiparametric studies can benefit from investments in machine learning.

As late as 2006, the argument was widespread, and is as yet unresolved, as to whether an increase in net photosynthesis can increase crop grain yield. Grain yield per unit ground area does not change much when panicle density or spikelets per panicle increase, because they are negatively correlated (Hasegawa *et al.*, 2013). Theoretically, increased photosynthesis can lead to increased grain yield and this has been observed experimentally under elevated atmospheric CO_2_. To translate potential increases in photosynthetic capacity into increased yield, other molecular factors or pathways may need to be engineered. Some recent scientific outputs that take trail-blazing routes to increase photosynthesis look very promising. For example, manipulating the photorespiratory pathway has led to exciting results for a biomass increase in tobacco (South *et al.*, 2019) and rice (Shen *et al.*, 2019). These and similar studies outlined in Table 1 provide the hope that it is possible to increase plant photosynthesis and see its effect on increased biomass and grain yield. However, for such studies to be useful for the future, it is vital to report on grain yield traits of high heritability. In the study by Shen *et al.* (2019), for example, the increase in rice grain yield was through the number of panicles per plant, which is a yield trait with very low heritability (Kato, 1997; Kamara *et al.*, 2017). The more dependable traits such as the 1000 grain weight and the number of filled grains per panicle were lower in transgenic plants engineered for the glycolate bypass compared with wild-type plants.

**Table 1.** Achievements and gaps in photosynthesis research towards crop yield increase

	Progress	Quality of evidence	References	Gaps
**Upstream**	Indirect selection	High	Zhu *et al.* (2008)	Efficient alleles
	Enzyme engineering	High	Kromdijk *et al.* (2016)	Genetic networks
	Mutant screening	High	Kirst *et al.* (2017)	Protein interactions
	Omics data	Medium	Wang *et al.* (2012)	Source–sink relationships
	Bioinformatics	Medium	Burgess and Hibberd (2015)	Stomatal mechanisms
	Process modelling	Medium	Kubis and Bar-Even (2019	Harvest index changes
	Gene cloning	High	Occhialini *et al.* (2016)	Trait heritability
	Gene editing	High	Khumsupan *et al.* (2019)	Nitrogen use efficiency
	Transgenic plants	High	Gu *et al.* (2015)	Carbon–nitrogen ratio
	Gene pyramiding	High	Kromdijk *et al.* (2016)	Biotic/abiotic stress
	Pathway engineering	High	South *et al.* (2019)	Microbiome changes
	Phenotyping	High	Keller *et al.* (2019)	Methane emission changes
**Translational**	Root exudates	Weak	Olanrewaju *et al.*, 2019	Role of rhizobiome
	Milling	Weak	Xu *et al.* (2015); Jing *et al.* (2016)	Grain quality
	Role of stomatal density	Weak	Way *et al.* (2014)	Scaled up analyses
	Biomass increase	Medium	López-Calcagno *et al*. (2019)	Commercial products
	Grain yield increase	Weak	Driever *et al.* (2017); Köhler et al. (2017)	
	Field test	Medium	Kromdijk *et al.* (2016)	
	Field phenotyping	Medium	Slattery *et al.* (2017)	
	Phenotypic changes in vascular bundle	High	Henry *et al.* (2017); Sedelnikova *et al.* (2018)	
	Carboxysome formation	High	Long *et al.* (2018)	
	Higher CO_2_ fixation	High	Yu *et al.* (2018)	
	FACE	Medium	Ainsworth and Long (2005); Gielen *et al.* (2005)	Extensive FACE studies
**Socio-economy and environment**				Market surveys
	Increased income	Weak	Klümper and Qaim (2014)	Cost–benefit analyses of alternative options
				*Ex ante* and *ex post* analyses
				Political economy of GE and seed systems
				Water use efficiency
				Land use efficiency
				Fertilizer/energy use efficiency
				Comparative technologies
				MEL processes
				Clear time scales of project termination

The ‘quality of evidence’ column reflects the overall body of evidence on the matter; it does not reflect the quality of the reference paper.

Notwithstanding the lack of definitive evidence for an increase in grain yield by manipulating photorespiration, the point about a steady relationship between high photosynthesis and high yield is nevertheless made through a reverse argument. For example, the ‘Green Revolution’ rice variety IR8, a high-yielding variety, had a greater photosynthetic rate per unit leaf area than previous varieties (Hubbart *et al.*, 2007). However, for rice, there is a biphasic pattern in variety development concerning photosynthetic efficiency. The varieties developed from 1966 to 1980 were better yielding due to better harvest index, while those developed after 1980 were better due to biomass increase (Hubbart *et al.*, 2007). In the latter case, the selection was most probably based on traits of high heritability. Hence, an increase in biomass still holds the promise of an increase in grain yield if the relevant yield traits are duly considered.

Overall, photosynthesis is still below its biological potential (Long *et al.*, 2006; Zhu *et al.*, 2008), and can be improved to increase yield potential, through an increase in either harvest index or biomass. In the former case, the source–sink relationship for assimilation, transportation, and accumulation plays a vital role; but the physiological mechanism for the latter case is still not clear. Similarly, the physiological and biochemical basis of very high photosynthetic efficiencies at high solar irradiance in the so-called ‘superperformers’ such as *Hirschfeldia incana* are not known, although some comparative genomics results have been presented (Garassino, 2018). However, does this high photosynthetic efficiency lead to high grain/seed yield? Not all highly photosynthetically efficient plants achieve high yield.

For FACE studies, there is an understanding that the increase in yield, can only be partly indicative of the natural scenario of the high CO_2_ future. These studies indicate less yield benefit than expected, but they report improved production over long time scales, even with suboptimal Rubisco activity; improved NUE and water use efficiency (WUE) and stimulated dark respiration (Leakey *et al.*, 2009). Studies taking other parameters of genotype, water, temperature, nutrients, and soil, and their interactions, into account under high CO_2_ are sparse. Since multivariable FACE studies are complex and expensive, modelling studies are used for insights and for informing field studies. However, comprehensive approaches that consider all the variables and their interactions do not yet exist. Degener (2015) used the BioSTAR crop model (https://www.uni-goettingen.de/en/431252.html) and considered different abiotic variables under two CO_2_ regimes to assess the quantitative and qualitative effect of CO_2_. Yield models were proposed over a century ago for 10 crops in a large agricultural area of Germany, spanning the maritime to continental climate. The study suggested that depending on the crop, season, and time scale of the model, one or more of the abiotic factors, or their interactions, become critical and lead to a reduction in crop yield. The beneficial effect of elevated CO_2_ may not be as high as predicted earlier, and whether CO_2_ levels in the future change in the predicted manner remains a key research question.

**Fig. 1.**
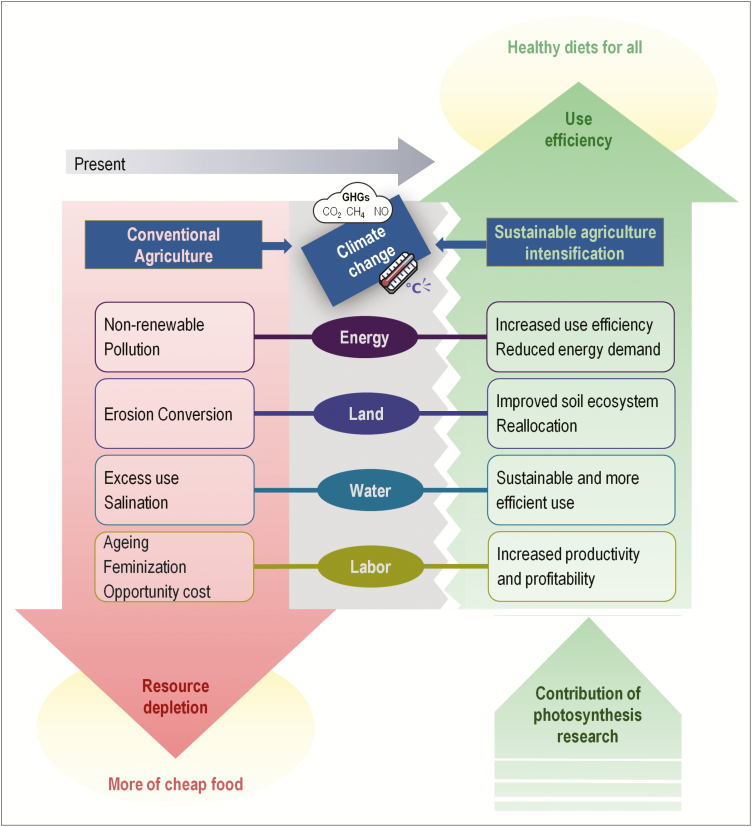
How can photosynthesis research support a paradigm shift toward higher food system efficiency? Conventional agriculture is causing incremental resource depletion. More land may be required to cater to future production scenarios. Land availability is constrained due to sprawling urbanization and other diverse land use. Agricultural soils are being eroded and depleted of minerals. Water is used indiscriminately and excessively. Heat- and drought-mediated, as well as seawater incursion-mediated, salinization of arable land restricts crop productivity. Excessive use of fertilizers and other chemical inputs also has consequences on pollution and energy consumption in a system that turns fossil fuel energy into food (Pimentel and Giampietro, 1994). At present, compared with the 1960s, more energy is needed to produce the same yield. Additionally, the working-age male population is leaving agriculture, leaving tasks in the fields to an older and female-dominated workforce. This scenario is compounded by the harmful effects of an anthropogenic climate change that is exacerbated by intensive agriculture. To break this paradigm, research for higher photosynthesis efficiency to obtain higher yields is one of the most promising avenues to develop more sustainable agricultural systems in the social, economic, and environmental contexts. Higher photosynthetic efficiency requires more carbon dioxide, which increases atmospheric carbon sequestration. Land systems re-balance by growing higher yielding crops which will free up land for other purposes. These crops will be more efficient in nutrient and water use, and be associated with a decrease in the labour to capital ratio. Similarly, reduced inputs will reduce the energy requirements from fossil fuels. Finally, higher yielding crops with high-efficiency photosynthesis and increased input use efficiency will increase productivity and reduce production costs, resulting in higher incomes for farmers. The additional income can be used for transformational changes such as child education, increased family welfare, and other necessities, such as better houses for better livelihoods.

Overall, the data to date support photosynthesis improvement as a plausible means to accomplish a quantum increase in crop yields. Yield increases at present are achieved by mechanisms that provide for more CO_2_ and/or improved WUE. In the future, increasing atmospheric CO_2_ concentrations may help increase the yields of some crops. Importantly, improved photosynthesis as seen under FACE studies portends improved water, energy, land, and labour use efficiency (Fig. 1). Such environmentally benign and sustainable multipronged benefits of improved photosynthetic efficiency, as opposed to the exponentially increasing environmental damage of contemporary agricultural practices, should elicit broad research interest in photosynthesis. The ambitious project aiming to convert rice from C_3_ to C_4_ photosynthesis is one example of global efforts in this direction.

## C_3_ versus C_4_ photosynthesis

An extensive body of literature exists to show that photosynthesis by plants such as maize, sugarcane, and sorghum, where the first product of carbon fixation is a four-carbon (C_4_) oxaloacetate, is more efficient than plants such as rice where a three-carbon (C_3_) 3-phosphoglycerate is first made. The efficiency resides in photorespiration-mediated recovery and concentration of CO_2_ within the plant cells (Sage *et al.*, 2012). The C_4_ plants can produce ~40–50% higher biomass and grain yields than C_3_ plants, due to the photosynthetic system that is 40% more efficient (Sage, 2016). Also, C_4_ plants have better NUE and WUE in the tropics and subtropics (Sage, 2016, and references therein). However, one advantage of C_3_ over C_4_ is the tolerance for shading, and that is a possible reason why C_3_ forests dominate over C_4_ plants (Sage, 2016). A modelling study by Gu *et al.* (2014) considered light-saturated and light-limited photosynthesis as key to scaling up the benefits of improved photosynthesis from leaf to canopy to crop level through genetic variation. Indeed the rich genetic diversity has not been queried for major differences in photosynthetic efficiency and there may be value in doing so. However, the question remains as to whether any of the unexplored rice that has higher photosynthetic efficiency will yield more than the top yielding rice varieties, because the purpose is to increase grain yield.

Most food crops are C_3_ plants; only maize, sugarcane, sorghum, and some millet are C_4_ plants that contribute to human nutrition. Therefore, with the potential of a much higher yield from C_4_ rice (Sheehy *et al.*, 2008), efforts to convert C_3_ to C_4_ plants by engineering the photosynthesis system may well be justified. Scattered attempts at trying to convert C_3_ photosynthesis food crops to C_4_ photosynthesis concentrated on introducing critical C_4_ genes (Häusler *et al.*, 2002; Miyao, 2003). For that, most researchers used the maize gene for phosphoenolpyruvate carboxylase (PEPC; Ku *et al.*, 2001; Taniguchi *et al.*, 2008). Due to the socio-economic importance of rice as a significant C_3_ grain crop, especially in the countries with food and nutrition insufficiency, it became a primary target from C_3_ to C_4_ conversion to increase productivity. Despite single-cell C_4_ mechanisms existing in aquatic and land plants to concentrate CO_2_ (von Caemmerer *et al.*, 2014), the two-cell C_4_ system (mesophyll and bundle sheath) is the route chosen for the conversion of rice from C_3_ to C_4_ photosynthesis. This conversion is a complex and ambitious process of manipulating not just the biochemistry, but also the anatomical features. It will take a large number of genes (~40 core genes) to achieve this in rice, and high-level transcription factors are suggested as primary targets to control numerous downstream target genes (Burgess and Hibberd, 2015). Apart from C_4_, other biochemical or physiological pathways have been suggested for improved photosynthesis (Skillman *et al.*, 2011). However, concomitant data on the effect on grain yield are lacking.

As costly and complicated as the C_3_ to C_4_ research may be, substantial financial and environmental gains are estimated once the final product is achieved (Sheehy *et al.*, 2008; Sage and Zhu, 2011). Additionally, engineering C_3_ into C_4_ plants provides a model for developmental and functional engineering of organisms in the future. After nearly a decade of the ambitious C_4_ rice project, a major concern is that intermediate C_3_–C_4_ products do not bring any benefit over C_3_ because the photosynthetic efficiency is less than or equal to that of C_3_ plants (Vogan and Sage, 2012; Heckmann *et al.*, 2014; Way *et al.*, 2014). Another setback has been the realization that knowledge of the molecular basis of C_4_ versus C_3_ photosynthesis is incomplete and cryptic, as highlighted by recent transcriptomic and bioinformatic data (Sedelnikova *et al.*, 2018).

One of the struggles of the C_4_ rice project has been the pressure to generate a product before the critical knowledge was available. Progress so far is therefore a combination of generating knowledge to fill the gaps in the mechanics of photosynthesis as a process, and harnessing available knowledge to develop the high-yielding product. For example, the catalytic turnover rate (*k*_cat_) and *K*_m_ for CO_2_ (*K*_c_) of Rubisco of C_3_ and C_4_ plants can be very different (Sage, 2002). The desirable high (*k*_cat_) Rubisco of C_4_ plants also has an undesirably high *K*_c_. Yet, the Rubisco of C_4_*Sorghum bicolor* has a higher *k*_cat_ to *K*_c_ ratio among C_4_ plants and may thus be a candidate gene to improve CO_2_ assimilation in rice (Ishikawa *et al.*, 2009). In the last decade, progress towards a better molecular understanding has been phenomenal, thanks to the interdisciplinary and global nature of the consortium. However, a spillover effect has been the recognition of the research gaps that remain, such as the mechanisms for optimal nitrogen partitioning. These gaps must be filled in parallel if we are to be ready to optimally use the gains from the upstream, fundamental biology progress in understanding photosynthesis *per se* and not just the differences in C_3_ and C_4_ photosynthesis (Table 1). Some further details on the scientific gaps are given below.

### Source–sink relationships

There has been much progress at the basic science level to understand how to improve the efficiency of photosynthesis. However, there remain gaps in how the additional assimilate must be transported and transformed into grain yield (source–sink and harvest index relationships). For example, there are numerous reports on genes, genomic regions, and genotypes for improved grain yield; most studies conclude that increasing leaf photosynthesis does not necessarily correlate to grain yield increase. However, there is a general agreement on the importance of the sink–source balance. The extent of changes in dry seed weight with changes in the amount of available assimilate in wheat, maize, and soybean suggests that grain yield is limited more by sink capacity than by the source (Borrás *et al.*, 2004). Thus, while photosynthesis increases source capacity, the limiting factor is in the sink (Borrás *et al.*, 2004; Long *et al.*, 2006; Takai *et al.*, 2011). Early seedling biomass determines later yield in many crops; therefore, by selecting for cultivars with desired sink and source traits, an increase in the rate of photosynthesis at early development stages leading to higher biomass can translate into higher grain yields as well (Long *et al.*, 2006; Ohsumi *et al.*, 2011; Takai *et al.*, 2013; Li *et al.*, 2014; Du *et al.*, 2019). The genetic component of such plasticity in grain yield, whereby an increase in source at the seedling or early vegetative stage can prime increased sink capacity, has been shown in a study with two soybean lines under high CO_2_ levels (Long *et al.*, 2006). Such plasticity in grain yield needs to be fully understood as a mechanism, and bred for, so that an engineered increase in photosynthesis under elevated CO_2_ is duly exploited and not limited by sink capacity. While the current drive to deliver the product quickly can address the issues of source–sink and harvest index relationships, other more critical gaps remain.

### Genetic and regulatory networks

The intricate genetic and regulatory networks underlying increased photosynthetic efficiency are still elusive. Are there superior alleles for the core set of enzymes? What are the molecular networks for associated processes with the capacity to influence photosynthesis, for example WUE? Local genomic regulatory hubs such as the one discovered through multigene quantitative trait loci (QTLs; Dixit *et al.*, 2015) can provide insights into sets of genes essential in such traits. Complex protein–protein interactions for how spatiotemporal alterations in protein products may engage with their known or new protein partners and how those, in turn, may affect the plant as a whole are yet to be fully understood. Nevertheless, this gap was known to exist, and an attempt was made to reconstruct the gene regulatory network for photosynthesis in Arabidopsis (Yu *et al.*, 2014), with little progress. In any case, the gene-, protein-, or metabolite-based information gaps may be filled through results from mega-projects such as the C_4_ rice project, where deeper molecular understanding is the mandate.

### Nutrient use efficiency

Despite clear evidence for improved WUE and nutrient use efficiency in C_4_ plants, there is little information on how improved C_3_ photosynthesis may affect resource use efficiency, including the carbon to nitrogen ratio and stomatal mechanisms.

Various factors influence the relationship between high photosynthesis and high grain yield, for example light response and genotypic variation (Long, 1993; Li *et al.*, 2014; Du *et al.*, 2019). Other factors of significant relevance when trying to increase yield by increasing photosynthesis are canopy architecture, leaf canopy size, NUE, and WUE. An increase in yield requires more water and nitrogen (fertilizer), and/or higher WUE and NUE (Mitchell and Sheehy, 2006). Several in-depth reviews of the plant nitrogen cost of photosynthesis, and nitrogen uptake and remobilization are available (Masclaux-Daubresse *et al.*, 2011; Evans and Clarke, 2019). C_4_ plants achieve a greater rate of photosynthesis for a given unit of leaf nitrogen because Rubisco operates close to CO_2_ saturation and less protein is invested in it. Leaf nitrogen in C_4_ plants is 20% lower than in C_3_ plants (Schmitt and Edwards, 1981; Sage *et al.*, 1987).

Yield gaps must be closed, to bring farmers’ yields closer to the assessed potential on experimental farms. Closing yield gaps can be achieved by increasing efficiencies of nitrogen and water, thus minimizing environmental impact associated with high demand, and through resilience to biotic and abiotic stresses (Simkin *et al.*, 2019). There is a trade-off through transpiration between biotic and abiotic stress tolerance and increasing yield by increased photosynthesis. Plants reduce transpiration as a mechanism to tolerate water stress. Increasing the rate of photosynthesis, and thus transpiration, would therefore reduce tolerance to stresses such as drought (Collins *et al.*, 2008; Driever and Kromdijk, 2013). Salinity-tolerant lines usually have reduced stomatal density, but this reduces photosynthesis and would potentially decrease crop yield (Liu *et al.*, 2017).

With NUE and WUE as essential components of harvesting the benefit of increased photosynthesis, what is our understanding of the relationships of root morpho-anatomy and physiology to altered photosynthesis? A recent study to increase WUE by genetic modification of PSII in tobacco found that light-regulated reduction of stomatal opening resulted in a reduction in water loss in the field (Głowacka *et al*., 2018). Although this discovery should increase productivity, the authors found that plant size and dry matter decreased in non-water-limiting conditions. It was hypothesized that protein PsbS excess and the concomitant increase in non-photochemical quenching level could adversely affect the light use efficiency under fluctuating light. An increase in nitrogen content could improve photosynthesis, which also affected stomatal conductance and chlorophyll amount, and the total nitrogen content in the leaves increased (Głowacka *et al*., 2018). These parameters were negatively affected under drought, but, even in water-limiting conditions, an extensive root system combined with an increase of nitrogen would permit more dry matter to be accumulated. The positive correlation between biomass accumulation, NUE, and photosynthetic rate suggests that greater NUE could be a useful parameter for drought tolerance (Dinh *et al.*, 2017). In their study, Saengwilai *et al.* (2014) reported a relationship between crown root number, nitrogen uptake, and increased photosynthesis.

### Rhizobiome

We understand little about how the rhizobiome is affected by C_3_ or C_4_ photosynthesis, or if the rhizobiome affects grain yield or quality. A recent paper by Olanrewaju *et al.* (2019) reported that there is variation in the types of exudates released into the rhizosphere of C_3_ and C_4_ plants. This supported the earlier research by Chen *et al.* (2016) who reported that changes in above- and below-ground biomass, and photosynthesis, would alter respiration in the soil through changes in substrate availability below ground. They conclude that C_4_ plants produce higher amounts of exudates and would provide more underground substrate for the respiration of both roots and the rhizobiome. While C_3_ plants exude more carbohydrates and organic carbons, C_4_ plants exude more organic acids and amino acids. Exudates of C_3_ plants are mannose, maltose, and ribose (Vranova *et al.*, 2013), and C_4_ plant exudates include inositol, erythritol, and ribitol (Olanrewaju *et al.*, 2019). C_4_ species would therefore favour bacterial respiration as the exudates are more abundant in labile materials, whereas C_3_ plant species would intensify fungal respiration as the exudates contain more stable compounds (Chen *et al.*, 2016). Nonetheless, the authors found that fungal respiration was predominant in both C_3_ and C_4_ species. Exudates of the C_3_ and C_4_ plants differ in pH and are hence mineralized differently (Tao *et al.*, 2004), most probably by a different rhizobiome. Some of the rhizobiome changes in C_3_ and C_4_ plants may be related to the communities that populate the various root systems and their functions. While ectomycorrhiza obtain carbon from plants and participate in carbon exchange between plants, endomycorrhiza exude sugars to the root system (Sasse *et al.*, 2018). Studies suggest that mutant plants that exude higher sugars than their wild types are more susceptible to disease. Therefore, alterations in the exudate composition of a particular crop could potentially have consequences on overall plant health and development. Greater understanding of plant–microbe interactions (e.g. substrate quality and quantity preferences) is needed to estimate the impacts of pests, pathogens, and tritrophic interactions on crops that exhibit altered photosynthetic efficiency. Plant root exudates are also more acidic from C_4_ plants (Tao *et al.*, 2004), and acidic soils are known for having fewer pathogens, fungi, nematodes, and beneficial bacteria (as cited in Lareen *et al.*, 2016). However, effects on the soil pH were minimal and thus the exudate changes between C_3_ and C_4_ plants may not have a major effect on root microbial communities. There have been studies of increasing seed yield by modifying the rhizobiome, but these alterations were related to specific inoculations known to apportion certain nutrients to the plant (e.g. the Sebacinales fungus in barley has been shown to increase yield and tolerance to stress, thus reducing yield losses; Waller *et al.*, 2005).

What would be the effects of rhizobiome changes to stress tolerance (pH, exudate composition)? We could not find studies that analyse the influence of the rhizobiome on grain quality or vice versa, other than the effects of adding biostimulants to the soil. Effects on root form and function, root exudates, and hence the rhizobiome can drastically affect the methane emission values, particularly of rice. With agronomic practices such as alternate wetting and drying and direct-seeding becoming popular, the reaction of the rhizobiome to such practices may or may not remain the same for plants with altered photosynthesis.

### Biotic stress

There is support in the literature for the attenuation of water-related abiotic stresses under elevated CO_2_ (Medina *et al.*, 2016; Li *et al.*, 2017) and increased photosynthesis (Yadav *et al.*, 2018), which might also positively affect heat stress (Shanmugam *et al.*, 2013), but there is little consideration for how the enriched biomass may react to the biotic stresses of pests and pathogens. On the one hand, there are reports that elevated CO_2_ can prime plant defences against fungal and bacterial pathogens (Mhamdi and Noctor, 2016). The adverse effect of elevated CO_2_ and temperatures on insect populations may be useful to plants, although altered feeding, fecundity, survival, and dispersal mechanisms may have a compensatory role (Trębicki *et al*., 2017). On the other hand, there are reports that increased biomass, due to increased photosynthesis, may be more prone to pests and pathogens (Ghini *et al.*, 2008). There is indeed evidence that reducing photosynthesis may be a useful defence mechanism against biotrophic pathogens (Garavaglia *et al.*, 2010). Importantly, biomass increase may not all be due to increased photosynthesis, and soil nutrients may have a critical role (Chatzistathis and Therios, 2013). In rice, in one study, an increase in biomass was seen to be inversely proportional to photosynthesis (Jahn *et al.*, 2011). There is a potential trade-off between abiotic stress tolerance and responses to biotic stresses, and further studies are required.

### Grain quality

No information could be found on how the improved photosynthesis, if it does translate into increased grain yield, may affect grain quality. These interactions should be modelled for better understanding because grain quality is critical for farmers and consumers. The rise in CO_2_ levels and the concomitant diminishing of photorespiration are both relevant for increased photosynthesis. However, climate change also comes with potentially adverse effects on crops, mainly through increased temperatures, decreased soil moisture, and a rise in phytotoxic tropospheric ozone (reviewed by Ort and Long, 2003; Long *et al.*, 2005). How such integrated scenarios may affect grain quality remains an important question. An increase in rice grain productivity generally leads to a decrease in quality (Anderson *et al.*, 1998; Dinh *et al.*, 2017). Superior grain quality in rice implies not only more protein, micronutrients, and fibre, but also a range of amylose and amylopectin ratios which depend on regionally preferred sensory parameters of cooking, taste, aroma, and palatability. In rice, grain quality also includes the extent of chalkiness which affects milling. Xu *et al.* (2015) reported that chalkiness increased and thus the milling qualities decreased with an increase in all three yield components, namely panicle number, the number of grains per panicle, and 1000 grain weight. Similarly, the amylose to amylopectin ratio and protein content suffer with higher yield and elevated CO_2_, as reported by Jing *et al.* (2016). Therefore, despite optimizing the source–sink relationships and obtaining higher yields, the product may have poor grain quality and not be commercially viable. For example, grain protein content is sensitive to O_3_ and CO_2_ concentrations in the atmosphere. When wheat grain yield was affected by 10% increases in O_3_ and CO_2_, the grain protein was 8% and 7.5%, respectively. O_3_ strongly negatively affects harvest index (Pleijel and Uddling, 2011), whereas it is unaffected by CO_2_ concentration. The direct negative response in wheat grain protein under elevated CO_2_ reinforces the hypothesis of abnormal nitrate uptake or assimilation (Pleijel and Uddling, 2011).

With protein content, grain quality suffered a reduction in the content of minerals such as iron and zinc under high CO_2_, as seen through a modelling study on mineral and protein deficiency diseases in women and children (Smith and Myers, 2018). Such a mineral reduction was predicted to have a drastic effect on hundreds of millions of people globally, with the most affected regions being South and South-east Asia, Africa, and the Middle East. There is increasing evidence of elevated CO_2_ leading to a decrease in the content of minerals and protein in the grain (Ujiie *et al.*, 2019). Thus, does a rice variety that has more efficient photosynthesis and a higher yield bear nutritionally inferior grains?

A newly developed rice variety, ‘Akiniwara’, is both high yielding and has good quality traits, particularly palatability. This variety has both an increased sink capacity and sink filling, demonstrating that these two traits are compatible. In their study, Yoshinaga *et al.* (2018) show that there was a minor reduction in perfect grain ratio related to increased sink capacity, and there was also a small increase in grain protein content compared with other high-yielding varieties and the control variety. There was also some evidence of lodging resistance in ‘Akiniwara’ (Yoshinaga *et al.*, 2018).

Concerns around the different aspects of grain quality are a starting point in considering the gaps in the downstream translational and socio-economic aspects of ensuring the research reaches the end-users. Perhaps in the absence of the end-product to date, the downstream gaps may not be pressingly relevant. However, apart from the commercial success, the effects on the environment, and the concomitant translational and transformational value of the product, other downstream considerations must also be taken into account in parallel (Table 2).

**Table 2.** Photosynthesis-related traits and pathways engineered for higher yield

Pathway/ experiments	Genes	Effects	Crop/Plant	Conditions	Productivity increase	Reference
Pathway 1: *Escherichia coli* glycolate oxidation Pathway 2: glycolate oxidase and malate synthase from plants and catalase from *E. coli* Pathway 3: Plant malate synthase and a green algal glycolate dehydrogenase (w/o down-regulation of a native chloroplast glycolate transporter in the photorespiratory pathway)	Undisclosed	Pathway 1: increased biomass ~13%. Pathway 2: same as wild type. Pathway 3: increased biomass by 18% (24% with RNAi)	Tobacco	Field	40% biomass increase	South *et al.* (2019)
TLA: truncated light-harvesting antenna	Su gene-aurea mutation (Su/su mutant)	Reduction of antenna size of the PS with decreased chlorophyll and carotenoids	Tobacco	Greenhouse	25% increase in stem and yield	Kirst *et al.* (2017)
TLA: truncated light-harvesting antenna	Reduced chlorophyll synthesis (YL mutant)	Smaller antenna size, reduced chlorophyll synthesis, higher thylakoid membrane proteins, increased PSII efficency, high electron transport rate, Rubisco activity and regeneration enhanced	Rice	Field and greenhouse	Similar yield in shorter growth duration. Higher yield in high plant density field conditions	Gu *et al.* (2015)
TLA: truncated light-harvesting antenna	Reduced chlorophyll synthesis (*Y11y11*, *y9y9* mutants)	Reduced chlorophyll content by 50%, increased photosynthetic efficiency and capacity early in the season. Capture less light and lower WUE by mutants impaired effects of lower chlorophyll under drought conditions suffered during the experiment	Soybean	Field	Same yield	Slattery *et al.* (2017)
Xanthophyll cycle and PSII	VDE-violaxanthin de-epoxidase ZEP-zeaxanthin epoxidase PsbS:PSII subunitS	Acceleration of NPQ relaxation and lower DES	Tobacco	Field	15% increase productivity	Kromdijk *et al.*, 2016
CO_2_ transporter system	Bicarbonate transporter BicA	BicA transporter localized 75% to thylakoind membranes and 25% to chloroplast envelope. Transporter did not show activity.	Tobacco protoplasts	Controlled conditions	NA Piloting stage	Pengelly *et al.*, 2014
CO_2_ transporter system	Bicarbonate transporters BicA and StbA	Targeting bicarbonate transporters BicA and StbA to the chloroplast inner envelope membrane	Tobacco	Controlled conditions	NA Piloting stage	Rolland *et al.* (2016)
Rubisco enzyme	Cyanobacterial Rubisco (*Gm-rbcL*, *Gm-rbcS*)	Cyanobacterial Rubisco expression enhanced in tobacco under high CO_2_ Higher carboxylation rates Need to introgress genes coding for vertex proteins and metabolite pore shells for fully functional carboxysomes	Tobacco	Controlled conditions	NA Piloting stage	Occhialini *et al.* (2016)
Carboxysome biogenesis	Carboxysome protein (*csoS1A*, *csoS2*) and cyanobacterial Rubisco (*cbbL*, *cbbS*)	Assembly of carboxysomes in higher plants to compartmentalize Rubisco and carbonic anhydrase	Tobacco	Controlled conditions	NA Piloting stage	Long *et al.* (2018)
Improve carbon fixation with malyl-CoA–glycerate synthetic pathway (MCG)	*Mcl* (malyl-CoA lyase), *gcl* (glyoxylate carboligase), *glxR* (or *GarR*) as tartronate semialdehyde reductase, *gark* (glycerate kinase I), *hyi* (hydroxypyruvate isomerase), and *ppc* (PEPC)	Assimilation of glyocoylate to produce acetyl-CoA Enhances bicarbonate assimilation by 2-fold	*Synechococcus elongatus*	Controlled conditions	Effects not measured on PS or yield	Yu *et al.* (2018)
Procambium formation/auxin pathway	PIN1, MP/ARF5, HD-ZIP III	Induction of vascular formation by auxin maxima. Auxin accumulation at convergence point, auxin flow, maintain meristematic competence in the procambial centre preventing new procambium formation in neighbouring cells	Arabidopsis	Controlled conditions	NA Proof of concept stage	Sedelnikova *et al.* (2018)
Radial patterning/SHR–SCR pathway	NAKED ENDOSPERM1, ZmRVN1, r ZmSCR1, ZmSHR1, OsSHR1, and OsSHR2	Specific pattern disposition of bundle sheath cells and mesophyll cells in the vascular bundles. Full characterization can take years.	Rice	Controlled conditions	NA Proof of concept stage	Henry *et al.* (2017); Sedelnikova *et al.* (2018)
Functionalization of vascular sheath cells	GOLDEN2, ZmG2-like1	Chloroplast development in bundle and sheath cells, chloroplast biogenesis. Induction of sustained development of chloroplasts in the sheath cells with subsequent chlorophyll increase	Rice	Controlled conditions	NA Proof of concept stage	Wang *et al.* (2017)

## Translational successes and gaps in photosynthesis research

Research on increasing the efficiency of photosynthesis requires extensive laboratory and field facilities and complex equipment, and genetic engineering can be an essential component. Hence, while studies in controlled greenhouses are the most common, there have been a few field studies. Some tests of laboratory-based yield and biomass increase have been taken into field conditions. A recent study on tobacco (López-Calcagno *et al*., 2019) demonstrated that yield increase is possible by manipulating photorespiration. The biomass increase relative to the control in the greenhouse was stable, but there was high seasonal variability in the field where the biomass increase was between 27% and 47%. By altering photorespiration, glycolate metabolism was again shown to increase tobacco crop biomass in field conditions by 40% relative to the control (South *et al.*, 2019). Although these are exciting developments, will the potential increase in biomass in cereals and other seed crops translate into an increase in grain yield? There are a few earlier studies in wheat (Driever *et al.*, 2017) and soybean (Köhler *et al*., 2017) where sedoheptulose-1,7-bisphosphatase was overexpressed to enhance photosynthesis and growth. The study on soybean focused on the effect of elevated CO_2_ on yield in the future climate scenarios, and an 11–22% increase in seed yield in the field was achieved (Köhler *et al*., 2017). In wheat, there was an increase in both biomass and grains, the latter being 40% higher than in the control under greenhouse conditions. The increase in the number of grains was through an increased number of grains per ear or increase in the number of ears being produced per plant (Driever *et al.*, 2017). These results validate the concept that, whether by elevated CO_2_ or by changing the mechanistic genetics, a change in photosynthetic efficiency can lead to increasing the grain yield. However, no enhanced varieties have crossed the pilot to commercialization barrier.

A few studies have looked at breaking the yield barrier in rice through altering photosynthesis and photorespiration, with minor success. One of these studies looked into the effects of co-overexpression of the genes of Rubisco and transketolase on photosynthesis in rice, which was greater by 35–53% and 39–84%, respectively, compared with the control (Suzuki *et al.*, 2017). The changes in protein content were related to alterations in the mRNA content. However, increased irradiance and different concentrations of CO_2_ did not alter the rate of CO_2_ assimilation between the control plants and those co-overexpressing the genes. Thus, in rice, the co-overproduction of Rubisco and transketolase did not improve photosynthesis. The overproduction of transketolase alone by 80–94% did not affect photosynthesis, suggesting that transketolase does not limit photosynthesis. A recent paper reported a new approach to boost photosynthesis in rice through the glycolate oxidase, oxalate oxidase, and catalase (GOC) bypass, which works by enriching chloroplasts with CO_2_ that otherwise would be lost during photorespiration (Shen *et al.*, 2019). The GOC bypass converts glycolate to CO_2_. Rice plants grown in the field showed higher photosynthetic efficiency, and increased biomass and nitrogen content. However, seed yield varied between seasons and even decreased when compared with control plants. Shen *et al.* (2019) claimed increased grain yield by increased panicle number, while the number of seeds per panicle and the 1000 grain weight was equal to, or less than, that of the plants not engineered in the glycolate metabolism pathway. Unfortunately, from the breeders’ perspective, panicle number per plant is a yield-related trait with the least heritability. Hence, one could argue whether the claimed grain yield increase in the transgenic plants is a stable trait.

Despite advances in research with novel strategies to increase grain yield in rice, the results are not conclusive and are somewhat disappointing for this crop. We still need more evidence about how the plant and components of the crop production and crop management system behave with modified photosynthesis because improving or altering one system has effects on other traits. For example, an increase in biomass in semi-dwarf rice varieties, which exhibit increased photosynthesis, led to an increase in the number of unproductive tillers, limiting the increase in yield (Khush, 2000). Questions remain around the trade-offs between photosynthesis increase and harvest index, NUE and WUE, the carbon:nitrogen ratio, and reactions to biotic and abiotic stresses.

An analysis of some high-yielding rice varieties, for example IR8, revealed that a high rate of photosynthesis was co-selected with high yield (Takai *et al.*, 2013). Additionally, experiments in rice showed that the sink could still be filled 50% more (Sheehy *et al.*, 2008). Therefore, increasing the source through photosynthesis in rice would not immediately be limited by the sink capacity *per se*. However, achieving the potential of the sink capacity is in turn dependent on other traits such as assimilate transportation and hormonal balances that facilitate assimilate transport, starch synthesis, starch component traits, and rate of dehydration.

Various studies identify related traits that support higher photosynthesis, such as thicker and greener leaves, stay-green top leaves, and flag leaf traits (Biswal and Kohli, 2013). However, an increase in biomass and photosynthesis (whether or not through C_4_) through higher leaf area would not necessarily provide either higher photosynthesis or higher yield, as the light intensity in the leaves would decrease due to canopy shading and the shade recovery processes. Alternatively, grain weight as a yield component is a trait of high heritability. It did not change much in the given varieties that showed increased yield under FACE study (Hasegawa *et al.*, 2013), suggesting that the emphasis should be on increasing panicle number and spikelets per panicle in the elite varieties. However, there exists large variability in grain weight, underpinning the importance of the multiple variables involved (Xu *et al.*, 2015; Li *et al.* 2019), and it can be positively or negatively affected in different genotypes, as shown in the case of wheat (Pleijel and Uddling, 2011; Broberg *et al.*, 2019). The relationship between increasing photosynthesis and yield remains unclear in many of the crops of importance to human food and nutrition security in the future, especially when other production factors are suboptimal. Even the effect of elevated CO_2_ on photosynthesis, biomass increase, and soil nitrogen supply remains to be set on a permanent footing through long-term studies because Reich *et al.* (2018) noted that trends observed as a response of C_3_ and C_4_ grasses to elevated CO_2_ reversed after 10 years. Their findings challenged the prevailing paradigms and suggested that short-term results may not be predictive of long-term effects.

## Potential socio-economic impacts and gaps in photosynthesis research

Research in upstream plant sciences is increasingly required to demonstrate its impact on people, the environment, the planet, and other species. How impactful has photosynthesis research been, or can be, for a farmer or consumer? Does an increase in photosynthesis add value towards creating viable and sustainable ecosystems? Building a strong case for photosynthesis research from these perspectives is more likely to attract resources. Ongoing upstream research on photosynthesis must be analysed to articulate a plausible impact pathway between investment and improved human and planetary prosperity.

Answering an array of questions may be necessary to demonstrate the socio-economic benefits of research on photosynthesis. The fundamental consideration would be the estimated net positive returns for farmers adopting the varieties with improved photosynthesis. This consideration, however, subsumes several questions. For example, in rice, can improvement in photosynthesis reduce farmers’ risk exposure? Can it reduce production costs? Can it generate premium grain quality traits, leading to the premium market price for the farmers? These questions, in turn, depend on dissemination and deployment strategies for the novel materials. For the most part, the programmes targeting material (technology) production and adoption tend to be rather linear and assume homogeneity of adopters. However, societies and economies are heterogeneous, and so are farm system vulnerabilities and exposure to risks (Lobell and Tebaldi, 2014). For example, remote locations limit access to markets (Barrett *et al.*, 2015), specific ecosystems limit access to natural resources, and restrictive economic surroundings and systems limit the capacity to thrive in increasingly sophisticated and complex cultivation and market systems (Reardon and Timmer, 2012; Béné *et al.*, 2019*a*, *b*). Hence, it is essential to consider policies that can maximize the positive effects, mitigate the negative consequences, and enable equitable distribution of additional wealth, especially among farmers. Farmers’ income can increase in three ways: (i) more production per unit and more sales at constant price; (ii) higher price per unit and/or savings through reduced input costs at constant volume; and (iii) change of the farming model by shifting to higher value crops or stepping out of farming altogether due to restrictive labour and input costs (Timmer, 1988; Dorward *et al.*, 2009; Reardon and Timmer, 2014; Barrett *et al.*, 2018). This third pathway is not relevant to the discussion on the socio-economic benefits of advances in photosynthesis.

The first option targets the production frontier. Constraints on land availability and the decreasing productivity of agricultural land (Huston, 2005; Mazoyer and Roudart, 2006) imply that increased production (and sales) must rely on filling the crop yield and labour productivity gap. However, unlike the Green Revolution paradigm of more food for more people on more land, today the market and social forces demand not just more food but also more nutritious food cultivated through sustainable and inclusive agricultural practices (Simelton *et al.*, 2012; Pingali, 2015). The hope is that advances in photosynthesis may lead to farm-level increases in ‘healthier’ yield at no or reduced cost to the environment. Figure 1 captures how more efficient photosynthesis contributes to improved ‘use efficiencies’ for critical resources that are being depleted or contaminated through conventional agricultural practices.

The second pathway to higher income suggests operating on, or even moving beyond, the price frontier, perhaps through innovations in input cost reduction. For example, adopting genetically engineered (GE) plants for herbicide tolerance can save the manual labour costs associated with weeding such that on the same piece of land the same volume of yield is now produced more cheaply, but the produce sells at the same market price. Barrows *et al.* (2014) studied the impacts of GE technology on agricultural supply and land use. They found that maize prices would have been 5–19% higher without cultivating GE crops, soybean would have been 19–33% higher, and cotton 9–17% higher. By increasing yields and reducing pest and weed control input costs, GE crop production helps control market prices. Reducing losses and waste during the production cycle also adds to the income.

Research on climate-smart technologies and practices (Campbell *et al.*, 2014; Steenwerth *et al.*, 2014) indicates that it is possible to increase input efficiency while also improving the socio-economic outcomes for farmers (Branca *et al.*, 2012; Karfakis *et al.*, 2012; ). The FACE studies of improved photosynthesis with elevated CO_2_ demonstrate improved NUE and WUE. Engineering more efficient photosynthesis under ambient CO_2_ concentrations may thus contribute to reducing such input costs. However, there is growing political pressure to re-internalize the externalities associated with intensive agriculture and there needs to be a more in-depth and holistic cost–benefit analysis. Thus, proper costing of a diminishing local water-table, poor soil and air quality, and loss of biodiversity associated with agriculture (Pingali, 2012) will inevitably translate into higher production costs (Benton and Bailey, 2019). Alternatively, as an example, reduction in one or more of the GHGs (CO_2_, CH_4_, and NO_2_) emissions resulting from more photosynthetically efficient plants could prove extremely valuable from both economic and environmental standpoints. Extensive modelling studies on the multiple parameters feeding into making increased photosynthesis successful must be complemented by socio-economic models to improve policy recommendations for impact, as recently exemplified by the resource-constrained scenarios (Kruseman *et al.*, 2020).

Since efficient photosynthesis can increase NUE, a parallel for fertilizer may be visualized by considering that between 1996 and 2014, herbicide and pesticide applications on GE crop fields were reduced by 8.2% (by active ingredient). The decrease in the Environmental Impact Quotient (Kovach *et al.*, 1992) indicator was 18.5% (Brookes and Barfoot, 2018). With fewer chemicals entering the environment, there is less contamination of water bodies and less damage to the environment and biodiversity (Mannion and Morse, 2012). Other environmental benefits arising from higher photosynthetic crop adoption include reduced fossil fuel use from application of fewer inputs and probably reduced soil cultivation contributing to lower GHG emissions (Brookes and Barfoot, 2018).

Techno-economic development, and especially the predicted decrease in the numbers of poor, small-scale farmers, calls for a clear vision on the ‘who, when, why, and how’ of the consumer of crops with improved photosynthesis. Product profiles should be the guiding principle for the next phase of photosynthesis research to ensure socio-cultural, environmental, and economic relevance of the final product. In effect, photosynthesis is probably the best understood basic biological process with potentially the highest likelihood of delivering a useful product. However, it is a highly complex process and we may spend another few decades only understanding it better, without heading towards and delivering a tangible product. Thus, using the process of photosynthesis to make a quantum difference in agricultural productivity has to be embedded in a multidisciplinary and holistic, yet pragmatic, analysis to guide large-scale projects. An approach that consists of starting with upstream sciences and later on exploring the field agronomy, markets, and political forces is far too linear. Instead, data on such downstream aspects should be generated in parallel to maximize the chances of final commercial success of the research product and that the required quantum leap in agricultural productivity is achieved sooner rather than later.

**Fig. 2.**
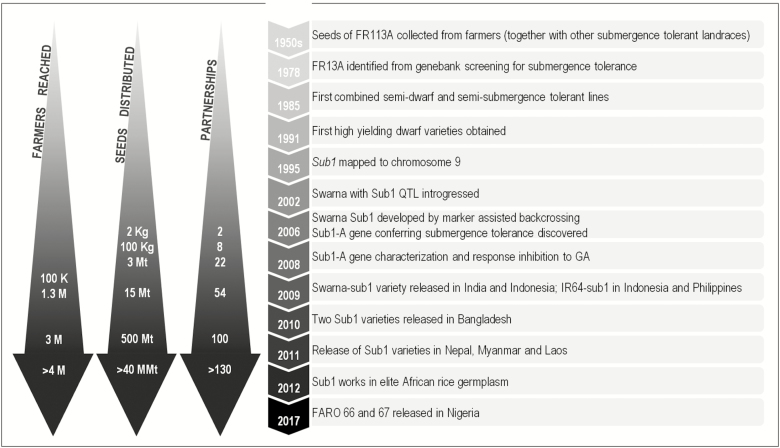
Timeline of the development of the Sub1 flood-tolerant varieties from experiment to scaling up to impact. As developments on the research aspect of the *Sub1* gene mechanistics and technology progressed, various advances evolved at the partnership and capacity development levels. Beyond scientific partnerships collaborating for product research and development, in 2006 National Agricultural Research and Education Systems were involved to scale up the seed dissemination capacity. Partnerships increased not only in number, but also in kind, and, by 2008, NGOs, farmer organizations, and private seed companies were also on board. Soon to follow, national government programmes and state governments, public and private seed companies, and international partners were also actively participating in extension, capacity building, and seed delivery systems. The enterprise was such a success that in 2013, 40 000 t of seeds were produced and 4 million farmers were adopting Sub1 varieties in various countries in South and South-east Asia. In the figure: kg=kilograms, T=tonnes, MT=‘000 tonnes, k=1000, M=million. After Bailey-Serres *et al.* (2010) and Mackill *et al.* (2012).

One good example to consider for how an integrative approach of parallel projects realized the vision of a product is submergence-tolerant rice, which is now a part of the farmer and customer base. Figure 2 shows a timeline for the development of the submergence-tolerant Sub-1 rice. It took nearly 40 years (1950–1990), since the identification of the tolerant phenotype in some rice accessions, to obtain a semi-dwarf, semi-tolerant rice line. However, within a little more than 20 years of the identification of the QTL, the downstream research and development could be achieved by involving a large array of stakeholders. Marker generation, marker-assisted backcrossing, marker-assisted selection, gene cloning, gene characterization, gene-based marker-mediated trait introgression, variety development, multienvironment testing, variety release, tolerant lines in elite local genotypes of multiple countries, seed dissemination in countries over three continents, and even impact analysis of the amounts of seed produced and number of farmers reached over time could be conducted thanks to an international collaboration network. Such progress was due to mega-projects involving multiple stakeholders right from genetics and genomics to local seed-distributing non-government organizations. The *ex post* analysis revealed an additional farmer income of nearly US$200 ha^–1^ (unpublished results). The additional income was used, among other expenses, to pay for children’s education—a transformative change. The important discriminator for success was not the search for a submergence-tolerant phenotype *per se* but for yield under submergence (David MacKill, personal communication). A similar approach of yield under drought has led to the identification of a number of large effect QTLs (Swamy *et al.*, 2011) some of which have now been pyramided (Kumar *et al.*, 2018), and the lines are a part of the breeding pipeline to develop drought-tolerant rice varieties. One of the QTLs has been extensively studied at the molecular level to highlight its complex functional nature (Dixit *et al.*, 2015; Raorane *et al.*, 2015*a*, *b*). The highlight in both submergence and drought tolerance research is the identification of QTLs for yield under stress. A similar approach for photosynthesis could target a panel of high- and low-yielding cereal genotypes with direct proportionality to photosynthesis (since there can be other avenues to yield increase; Jahn *et al.*, 2011). With an extensive array of molecular–physiological understanding, available omics data, and the advantage of an expert upstream research consortium already operative, any quantitative genetics discovery could be quickly exploited for characterization and further utilization in the breeding pipeline. Hence, the two main factors contributing to success would be the investment in collaboration with downstream breeders, agronomists, and socio-economic experts, and the identification of clear time- and stage-bound deliverables to allocate resources optimally. For example, if no strong QTLs could be identified soon, it would be an indication that there is not much allelic variation in the genes involved. Such a result may suggest the need for higher order mechanisms such as the protein post-translational modification or protein–protein interaction networks. Simultaneously, physiologists and agronomists could be looking at the same diversity panel for clues to suggest target mechanisms such as NUE, WUE, and source–sink relationships to be more deeply explored. The product profile data for target eco-geographies, policy research, and *ex ante* socio-economic surveys would provide information on the scope and target scenarios for success of the product in the pipeline in order to maximize the impact of research. Collaboration with groups at the country level would take the product through local trials and prime the seed sector and other crucial partnership, while at the same time involving governments and other local organizations.

## Concluding remarks

Despite incremental evidence for its potential, research on mechanistically improving photosynthesis efficiency is still insufficient to ensure its value in creating more grain, seed, or fruit yield. Recent literature shows there needs to be an assessment of the pros and cons of improving the single or dual cell photosynthesis systems. Unlike the ‘Green Revolution’, technologies for increasing agricultural productivity now must factor in additional economic, social, cultural, and political aspects to ensure success. More importantly, whether it is converting C_3_ crops into C_4_ crops, or only increasing the photosynthetic efficiency of C_3_ plants, a parallel multistakeholder approach is more likely to succeed. The information on the mechanisms and pathways of photosynthesis, gained especially in the last decade when the upstream photosynthesis science is conducted with a vision of the downstream product, allows scientists to understand better how to mine our genetic resources to improve crop yields. It is also critical to assess if the elevated CO_2_ of the future may be the source for highly efficient photosynthetic plants, what the life cycle assessment route for the planetary boundaries would be in the altered eco-system, and how that would be sustainable. Now is the time to consider what the real benefits will be, and to whom they may accrue. There are apparent trade-offs, most obviously in what is known to be physiologically connected to photosynthesis improvements such as the NUE and WUE. How can these be balanced but, more importantly, are these the only connected parameters? Could other perturbations render the advancements commercially non-viable, such as grain quality, root structure, and function, rhizobiome, biotic and abiotic stress responses, etc.? Will grain quality diminish under a high CO_2_ and/or high temperature climate? How does that compare with a CO_2_ increase mechanistically within the plant cells? This information is not currently available. How does the present state-of-the-art in gene editing, with respect to technologies and regulatory needs now and in the future, factor into the acceptance or otherwise of the GE crops? Hence, what timelines and boundaries exist to assess the viability of current or future technical approaches? How valid and valuable is the translational value of the resources and efforts spent upstream? We cannot wait to have the full knowledge and understanding of an increasingly complex food system to start tinkering with it, but equally we do not have the luxury of continual research without linking it to socio-economically feasible and acceptable products.

Increased photosynthesis primarily translates into biomass increase but may increase grain or seed yield. Where the biomass is the product, the chances of success are high. Success with conventional mechanisms to further increase production in the field has, to some extent, plateaued. Based on FACE studies, a breakthrough seems plausible from the modification of photosynthesis by GE mechanisms. Such a breakthrough could have both positive and negative effects on the farmers as they should expect, for this increased productivity and profitability, to pay higher prices for seed of GE varieties as a result of the cost of stringent stewardship; they will need to become more vertically integrated to obtain GE seeds and may need to apply more fertilizer (mostly nitrogen) to feed the increased biomass.

Breakthroughs in upstream research such as improved photosynthesis need to be understood and assessed in the broader context. The success underpinning the ‘Green Revolution’ in improving the farmers’ livelihoods (Hazell, 2009; Pingali, 2012) is embedded not only in the improved plants, but also in the use of recommended crop management practices, increased use of fertilizer inputs, and improvements in infrastructure made by governments (Smale, 2016). Unfortunately, many farmers of the ‘Green Revolution’ have remained poor because at that time ‘poverty disaggregation’ was not considered broadly. Thus, improved photosynthetic research requires a phased, yet parallel, integrated approach, not just at the level of multidisciplinary science teams, but at the level of multiple stakeholders in both the public and private sectors. It needs to ensure that the breakthroughs from upstream photosynthesis research can translate into the form of increased crop productivity, more stable farm gate prices, increased farmers’ income, as well as improved consumer satisfaction.

Internalizing and transferring such technologies and products into national agricultural systems cannot happen without additional investments in both upstream and downstream research. Such direct participation in dissemination or deployment is necessary with the proactive review and negotiations for international legal instruments that facilitate an equitable distribution of financial value embedded in novel intellectual property or existing traditional knowledge (Rao, 2017). Public–private platforms show the way forward for the governments of developing countries to act in the interest of resource-poor farmers.

## Abbreviations

FACE: free-air CO_2_ enrichment
GE: genetically engineered
GHG: greenhouse gas
NUE: nitrogen use efficiency
PEPC: phosphoenolpyruvate carboxylase
WUE: water use efficiency
